# Hemodynamic parameters impact the stability of distal stent graft-induced new entry

**DOI:** 10.1038/s41598-023-39130-5

**Published:** 2023-07-26

**Authors:** Kaihong Wang, Chlӧe H. Armour, Tao Ma, Zhihui Dong, Xiao Yun Xu

**Affiliations:** 1 Department of Chemical Engineering, Imperial College London, South Kensington Campus, London, SW7 2AZ UK; 2 Department of Vascular Surgery, Zhongshan Hospital, Fudan University, Shanghai, China

**Keywords:** Biomedical engineering, Aortic diseases

## Abstract

Stent graft-induced new entry tear (SINE) is a serious complication in aortic dissection patients caused by the stent-graft itself after thoracic endovascular aortic repair (TEVAR). The stability of SINE is a key indicator for the need and timing of reinterventions. This study aimed to understand the role of hemodynamics in SINE stability by means of computational fluid dynamics (CFD) analysis based on patient-specific anatomical information. Four patients treated with TEVAR who developed a distal SINE (dSINE) were included; two patients had a stable dSINE and two patients experienced expansion of the dSINE upon follow-up examinations. CFD simulations were performed on geometries reconstructed from computed tomography scans acquired upon early detection of dSINE in these patients. Computational results showed that stable dSINEs presented larger regions with low time-averaged wall shear stress (TAWSS) and high relative residence time (RRT), and partial thrombosis was observed at subsequent follow-ups. Furthermore, significant systolic antegrade flow was observed in the unstable dSINE which also had a larger retrograde flow fraction (RFF) on the SINE plane. In conclusion, this pilot study suggested that high RRT and low TAWSS may indicate stable dSINE by promoting thrombosis, whereas larger RFF and antegrade flows inside dSINE might be associated with its expansion.

## Introduction

Stent graft-induced new entry tear (SINE) is a serious complication after thoracic endovascular aortic repair (TEVAR) for aortic dissection (AD). Although SINE could remain stable in some cases, it may be more dangerous than the untreated AD^1–3^, since SINE does not have an outlet and eventually requires a secondary aortic repair to prevent rupture^4–7^. Due to the severity of the potential complications and an up to 25% incidence of SINE^1,8,9^, there is a need to develop an efficient risk stratification method for SINE.

The risk factors leading to SINE formation have been investigated in several recent studies. Strong radial force from stent graft in the proximal landing zone (designed to strengthen the proximal fixation and prevent stent migration) has been reported as the cause of retrograde type A dissection^1,10^. Distal stent-graft oversizing and mismatch between the stented and non-stented regions are other identified risk factors. The radial force exerted on the aortic wall by the stent graft is distributed transversely but not longitudinally, resulting in better remodelling in the stented region but less prominent remodelling in the non-stented regions^8,11^. Moreover, oversizing at the distal end of stent graft can lead to large spatial variations in wall shear stress around the junction, increasing the risk of dSINEs^8,11,12^. From clinical studies, an average distal oversizing ratio of 230% by area was observed in SINE patients, which was much higher than in non-SINE patients with an average value of 130%^8,13^. The descending thoracic aorta taper ratio, which measures the difference in diameter between the aortic arch and the distal true lumen, was also found to be high in patients who underwent reinterventions^8,14^, but the role of taper ratio was questioned by other researchers^13^.

Computational studies have been carried out to investigate risk factors related to the formation of SINE. By performing finite element analysis of wall stress in the aortas of dSINE and non-SINE patients, Menichini et al. found higher wall stress at the distal end of the stent graft on the dSINE patient, and suggested that higher stent graft tortuosity may be a risk factor for dSINE^15^. The effect of stent graft tortuosity was further investigated by Tan et al. who showed that increasing stent graft tortuosity from 50 to 30° could elevate the maximum von Mises stress from 263 to 313 kPa^16^. A recent study by Osswald et al. examined the role of wall shear stress (WSS) in the occurrence of dSINE on 5 patients, and they found elevated WSS was a potential risk factor for adverse events, especially in regions close to the end of the stent graft^17^. However, another computational study by Qiao et al. found that WSS was weaker in predicting the deterioration of dSINE^18^. In addition, Ma et al. investigated the effect of oversizing ratio on the occurrence of RTAD, and their finite element analysis results showed that increasing aortic diameter oversizing ratio from 0 to 15% resulted in more than two-fold increase in the maximum von Mises stress (from 0.51 ± 0.07 to 1.32 ± 0.74 MPa) in the proximal landing zone^19^.

After the formation of a SINE, its progression will affect the choice of further treatment, and the stability of SINE is a key indicator for the timing of additional interventions. Rapid expansion of the SINE and contained rupture require timely re-interventions, while a stable SINE is usually treated with medical management initially. From a retrospective study by Weng et al., the average time period between the first detection of dSINE and reintervention was 26 months^8^. It remains unclear at the time of detection whether a SINE will remain stable or not, and thus there are risks in waiting for clinical indications to perform reinterventions. In this regard, computational studies may help identify risk predictors for unstable dSINEs. To the best of our knowledge, computational studies seldomly focused on the stability of dSINE and little is known about the hemodynamic conditions after dSINE formation.

The present study aims to investigate the influence of hemodynamics on the stability of dSINEs. Computational fluids dynamics (CFD) simulations were performed on 4 dSINE patients (2 stable and 2 unstable dSINEs). Time-averaged wall shear stress (TAWSS), relative residence time (RRT), and retrograde flow fraction (a parameter identified in previous studies to correlate with false lumen expansion^20^) at different locations were compared among the patients to identify potential predictors of dSINE expansion.

## Results

### Anatomical characteristics

**Figure 1:**
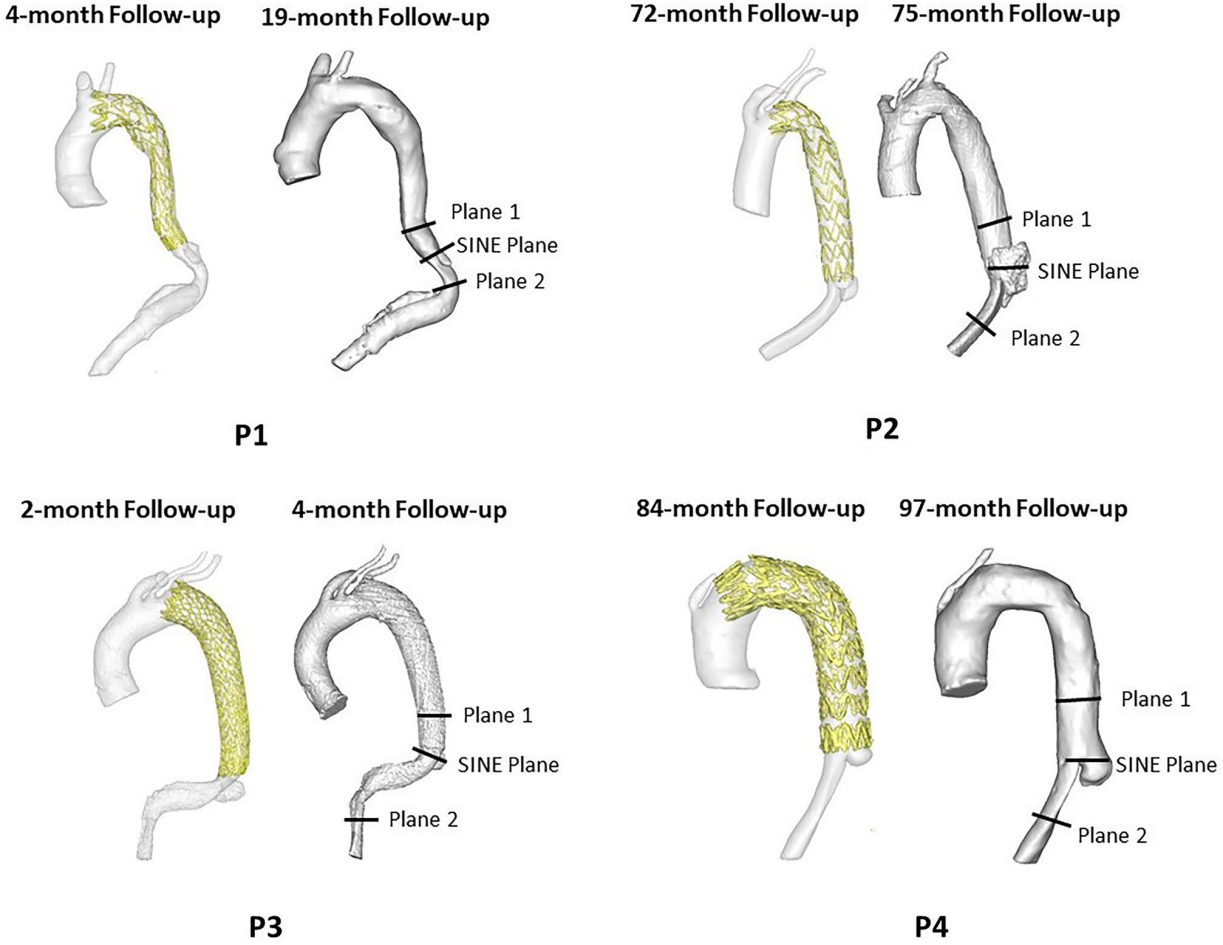
Reconstructed geometries based on CT scans at follow-ups of early and post dSINE. P1 and P2 presented a stable dSINE; P2 and P4 presented an expanded unstable dSINE.

The SINE volume and the percentage change between the first detection of the SINE and the first post-SINE follow-up scans are reported in Table 1. The dSINE contracted in P1 and P3, with a volume reduction of around 50%. In P2 and P4, the dSINE expanded and its volume increased by 292.5 and 23.0%, respectively (Fig. 1).

**Table 1:** Volume measurements of dSINEs.

	V*SINE*, mm^3^	V*post* − *SINE*, mm^3^	Difference (%)
P1	1924.4	956.5	− 50.3
P2	4800.9	18,842.4	292.5
P3	5325.4	2717.1	− 49.0
P4	3413.9	4198.8	23.0

*V*_*SINE*_: the volume of SINE at first detection on CT scans; *V*_*post*_ − _*SINE*_: the volume of SINE at the first post-SINE follow-up.

### TAWSS and RRT

**Figure 2:**
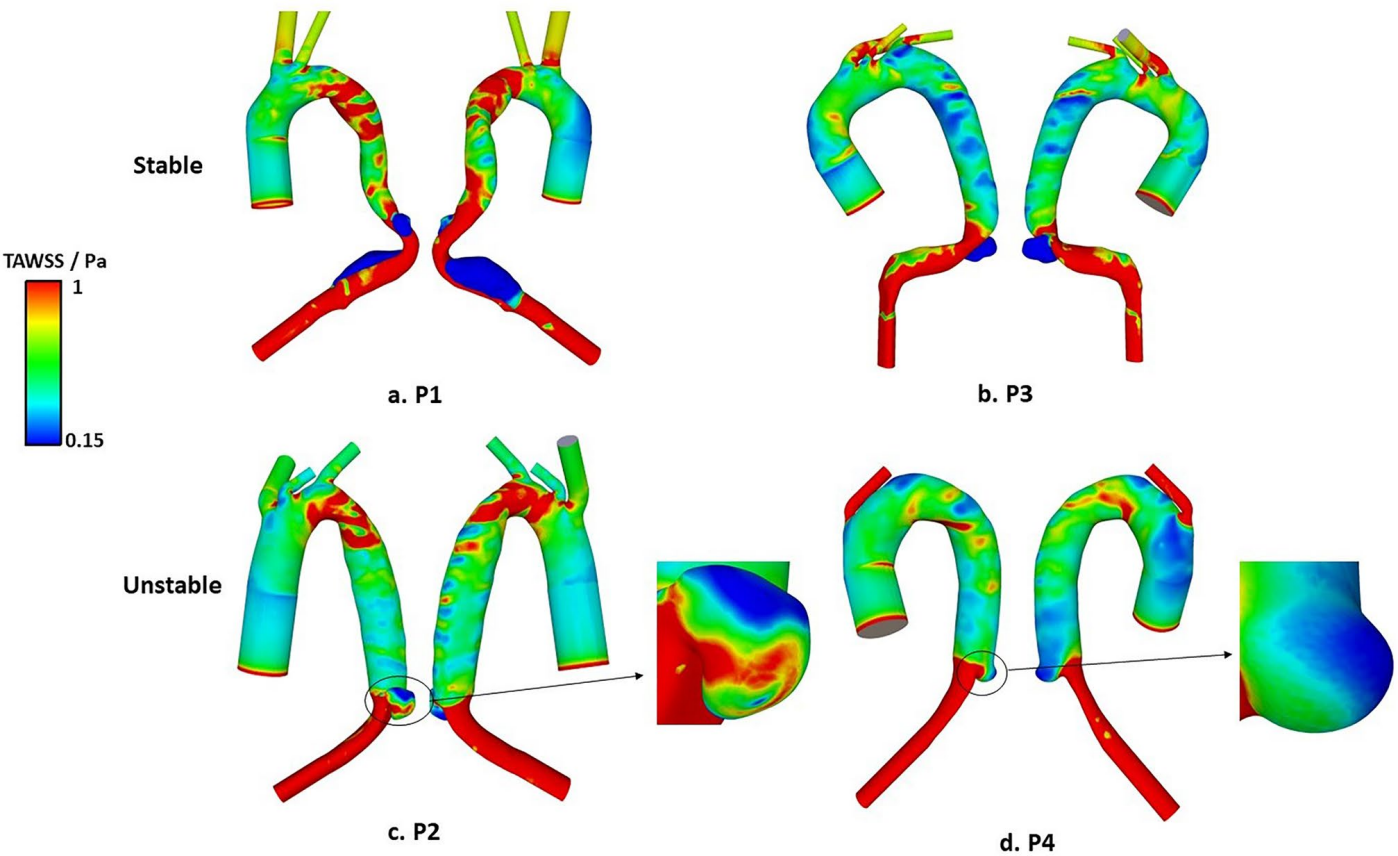
TAWSS distributions in all simulated models. (**a**, **b**): Low TAWSS was observed on the entire surface of the stable dSINE; (**c**, **d**): Unstable dSINE presented a large region with elevated TAWSS (> 0.75 Pa).

Figure 2 shows the predicted TAWSS contours for all four aorta models with dSINE. Lower TAWSS was observed in the ascending aorta in all models and in the stented region of the thoracic aorta in P2, P3 and P4. The distal section of the descending aorta below the stent graft was characterised by elevated TAWSS. In the SINE region, average TAWSS values were calculated, and the results are shown in Table 2. P1 and P3 presented extremely low TAWSS, while P2 and P4 had higher TAWSS compared to P1 and P3.

**Table 2:** Values of average TAWSS and RRT in the SINE for P1, P2, P3 and P4.

	Average TAWSS_*SINE*_ (Pa)	Average RRT_*SINE*_
P1	0.17	33.4
P2	0.62	3.5
P3	0.06	87.5
P4	0.42	6.7

TAWSS_*SINE*_: Time-average wall shear stress on the SINE surface; RRT_*SINE*_: Relative residence time on the SINE surface.

**Figure 3:**
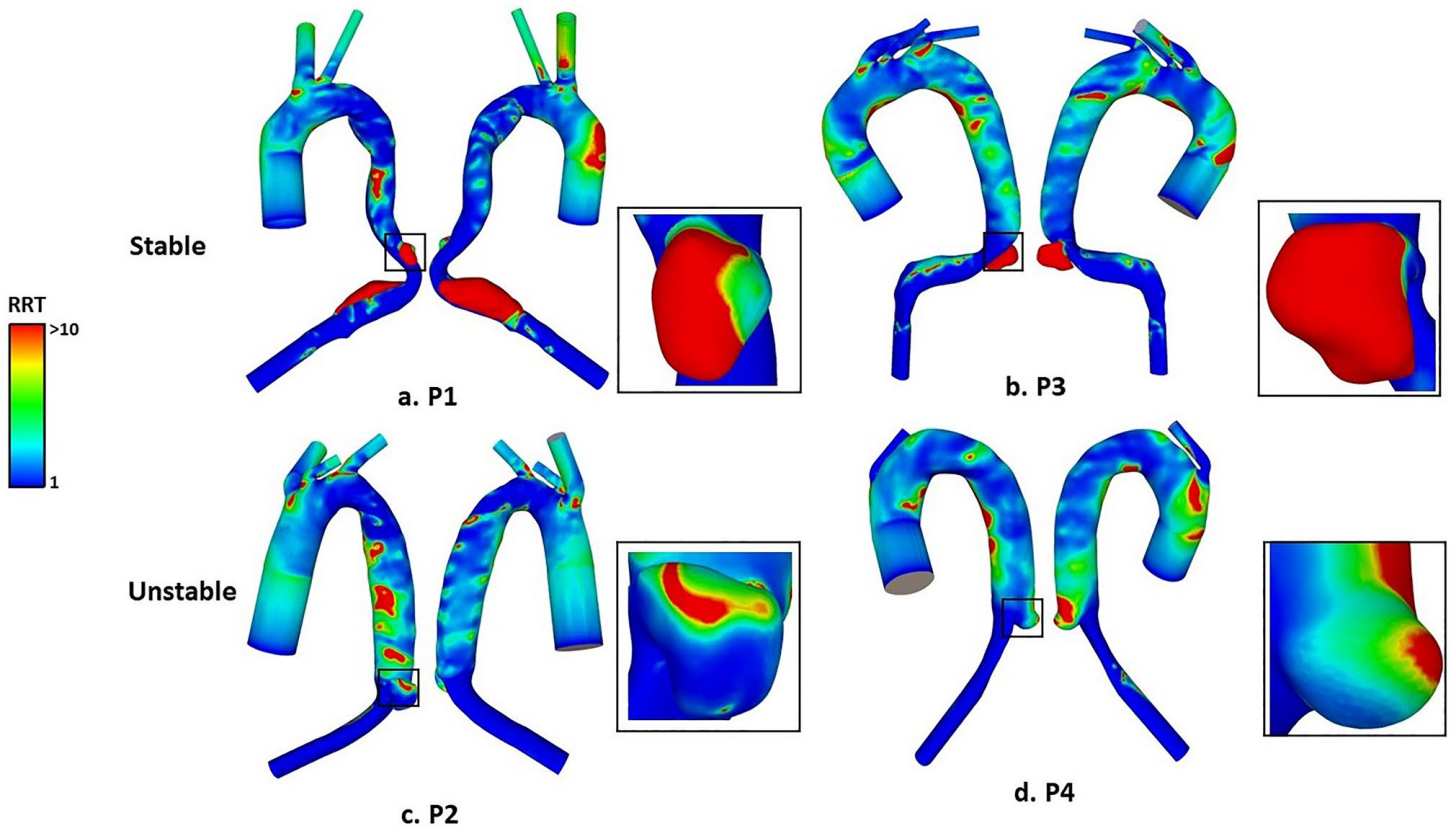
RRT distributions in all simulated models. (**a**, **b**): High RRT (> 10) was observed on the surface of the stable dSINEs. (**c**, **d**): On the surface of the unstable dSINE, low RRT was generally observed.

Figure 3 shows RRT distributions for all four models. In P1 and P3, high RRT was observed on the SINE surface, while in P2 and P4, a large part of the SINE region experienced low RRT compared to P1 and P3. Average values of RRT on the SINE surface were calculated and the results are also included in Table 2.

### Flow patterns

**Figure 4:**
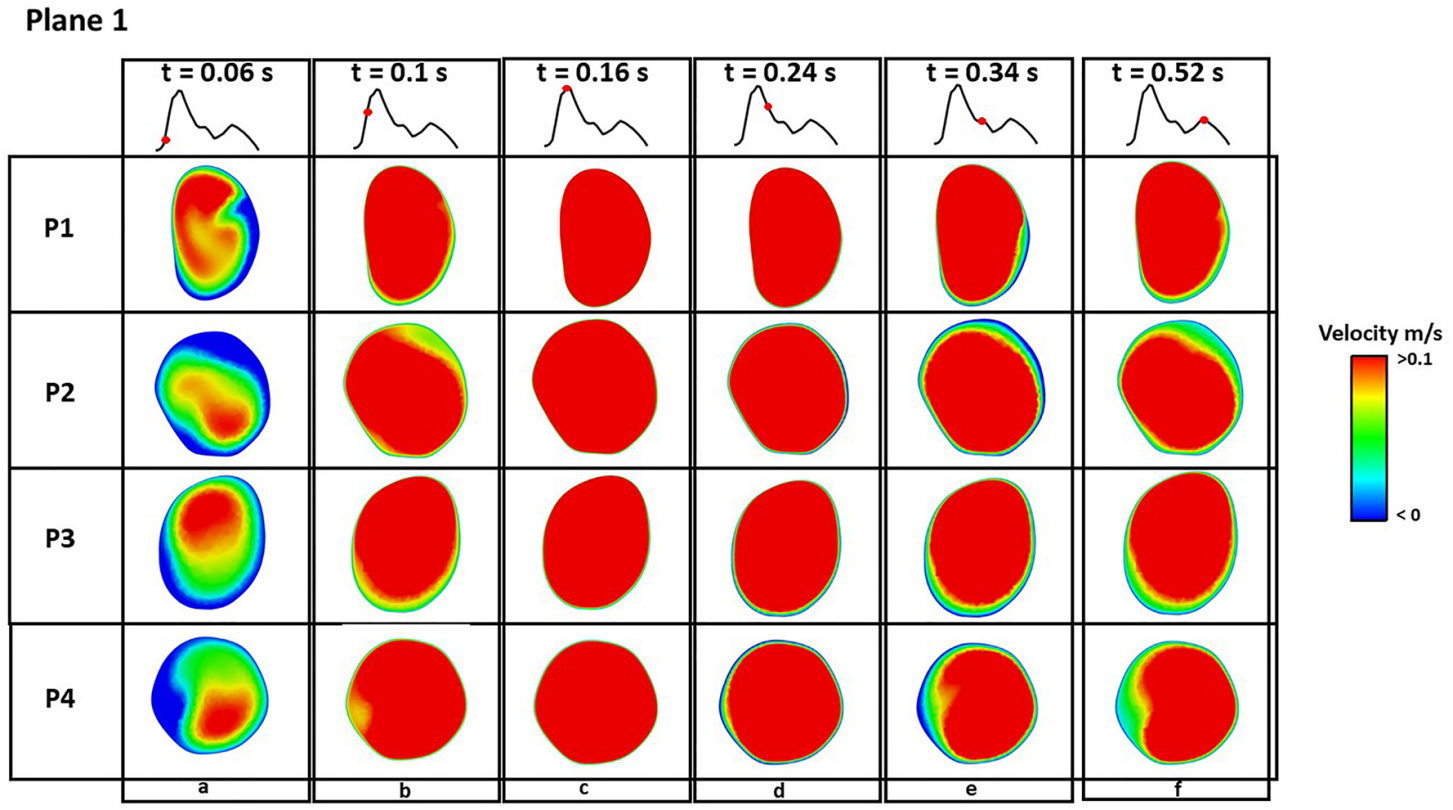
Velocity contours on plane 1 at various timepoints throughout a cardiac cycle. Negative velocities represent retrograde flows. Retrograde flow is observed in P1, P2, P3 and P4 at early systole t = 0.06 s, and nearly no retrograde flow is observed during the rest of the cycle.

**Figure 5:**
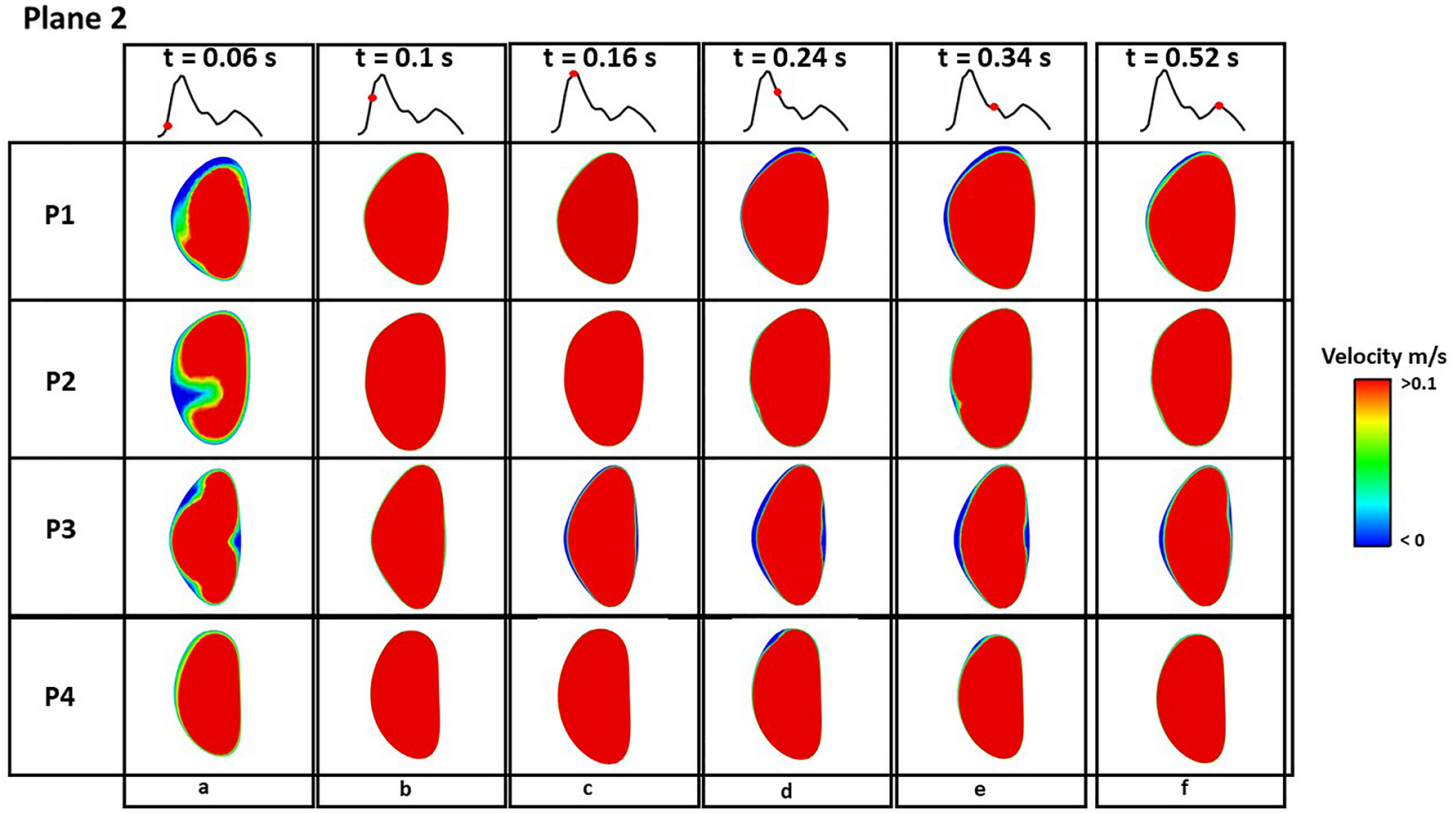
Velocity contours on plane 2 at various timepoints throughout a cardiac cycle. Negative velocities represent retrograde flows. More retrograde flow is observed in P2 compared to P1, P3 and P4 at early systole t = 0.06 s, and nearly no retrograde flow is observed during the rest of the cycle.

**Figure 6:**
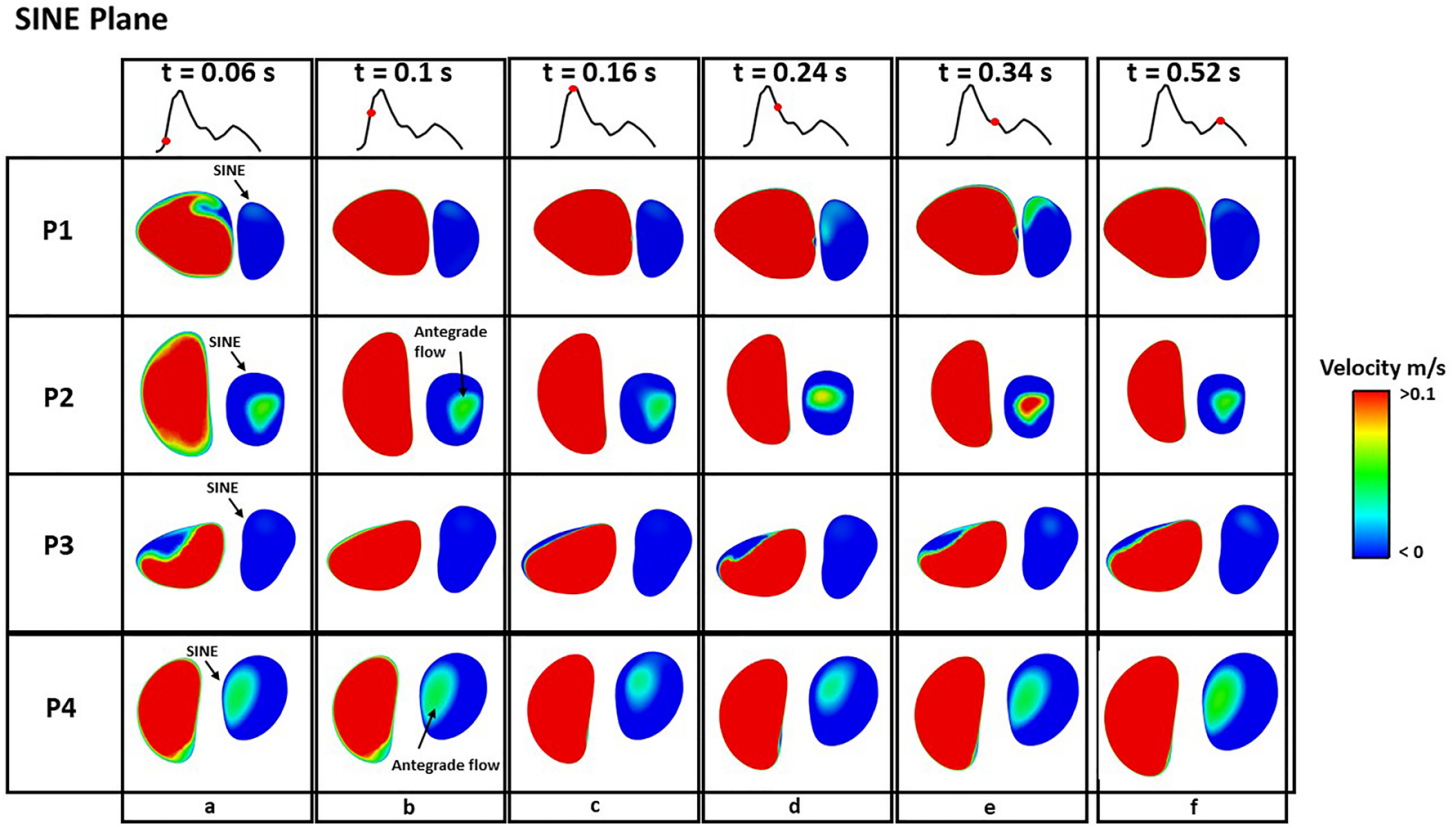
Velocity contours on SINE plane at various timepoints throughout a cardiac cycle. Negative velocities represent retrograde flows. Antegrade flow is observed in the TL in P1, P2, P3 and P4 at all timepoints. In the SINE, P2 and P4 present a large region with antegrade flows, but these are not observed in P1 and P3.

Figures 4 and 5 show instantaneous velocity contours on plane 1 and 2 (defined in Fig. 1), respectively, where retrograde flows are represented by negative velocities (blue colour). At early systole (Fig. 4a and Fig. 5a), retrograde flow was observed on both planes in all patients, and a larger region with retrograde flow was observed in P2. Throughout the remaining systolic phase, effectively no retrograde flows were observed on either plane in all patients (Figs. 4b–d and 5b–d). During diastole, more retrograde flows were observed at the edge of plane 1 in P2 and P4 (Fig. 4e, f), but this difference was not obvious on plane 2 (Fig. 5e, f). In addition, velocity contours on the SINE plane at different timepoints are shown in Fig. 6. In the true lumen (TL), retrograde flow was observed in P1 and P3 at early systole (Fig. 6a), which was not obvious in P2 and P4. In SINE, P2 and P4 presented antegrade flows in the centre of the SINE plane throughout the cardiac cycle, but no antegrade flows were observed in P1 and P3.

**Figure 7:**
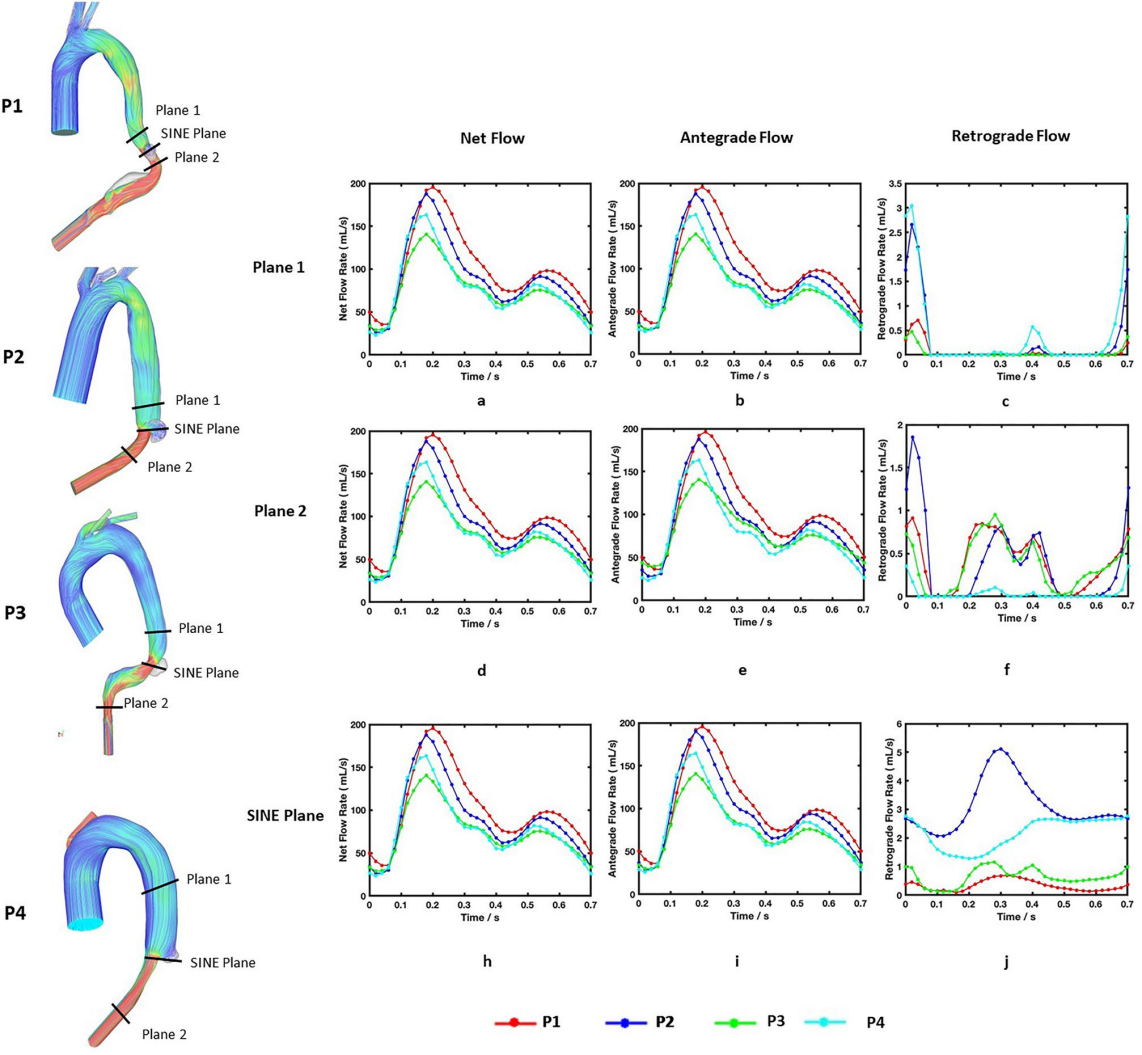
Flow measurements on plane 1 and 2 and SINE plane over a cardiac cycle. (**a**, **d**, **h**): Variations of net flow; (**b**, **e**, **i**): Variations of antegrade flow; (**c**, **f**, **j**): Variations of retrograde flow. P3 had lower net and antegrade flows due to a smaller tear size, and more retrograde flow was observed in P2 and P4. On the SINE plane, significant difference was observed between stable and unstable dSINEs.

The net flow, antegrade flow, and retrograde flow were measured on each plane, and their variations over a cardiac cycle are shown in Fig. 7. Similar trends were observed in both net and antegrade flows, although P3 had lower peak values due to a smaller tear size. On plane 1, retrograde flows occurred in all four patients, but higher level of retrograde flow was observed at early systole in P2 and P4 (Fig. 7c). On plane 2, retrograde flows were observed during most of the cycle in all models (Fig. 7f). Values for RFF during systole and diastole were calculated based on Eq. (5) and summarized in Table 3. P2 and P4 had more retrograde flow on plane 1 and the SINE plane during both systole and diastole, while there was little difference on plane 2. On SINE plane, it can be clearly observed that there was more pronounced difference in retrograde flow. Quantitative comparisons of RFF, *SINE*_*SA*_ and *SINE*_*DR*_ in Table 3 showed that P2 and P4 had larger RFF during both systole and diastole and larger *SINE*_*SA*_ compared with P1 and P3.

**Table 3:** Flow measurements: retrograde flow fraction (RFF), systolic antegrade SINE flow fraction, and diastolic retrograde SINE flow fraction.

	Systole RFF			Diastole RFF			SINE_*SA*_ (%)	SINE_*DR*_ (%)
	Plane 1 (%)	Plane 2 (%)	SINE plane (%)	Plane1 (%)	Plane 2 (%)	SINE plane (%)		
P1	0.08	0.39	0.25	0.03	0.43	0.39	0.21	50.18
P2	0.35	0.46	2.63	0.26	0.46	4.37	1.14	50.64
P3	0.06	0.45	0.66	0.06	0.57	1.07	0.23	62.34
P4	0.61	0.61	1.72	0.45	0.45	3.49	1.74	49.96

RFF: Retrograde flow volume fraction with respect to net flow volume; On SINE plane, RFF is calculated using total flows (true lumen + SINE). SINE_*SA*_: Systolic antegrade SINE flow volume (SINE only) fraction with respect to total (true lumen + SINE) systolic antegrade flow; SINE_*DR*_: Diastolic retrograde SINE flow volume (SINE only) fraction with respect to the total (retrograde + antegrade) diastolic SINE volume.

## Discussion

The implantation of a stent graft leads to local geometric discontinuity and material property mismatch between the stented and non-stented regions, and these can result in large spatial variations in stress distribution. The location of stress concentration has been found to increase the risk of SINE formation^19,21^. Additionally, anatomical features, such as stent graft tortuosity and distal oversizing ratio, play an important role in determining stress distribution^15,16^. However, once a SINE is formed, how it will develop, or its stability will cause different clinical outcomes and affect the timing of additional intervention. It would be desirable to identify hemodynamic or biomechanical factors that determine SINE stability, so that patients at high risk of SINE expansion would benefit from early reintervention. The application of CFD to patient-specific geometries may help elucidate the role of hemodynamics in the long-term development of SINE. To the best of our knowledge, only two recently published studies reported flow analysis in dSINE following TEVAR of aortic dissection^17,18^, and more data is clearly needed in this respect.

**Figure 8:**
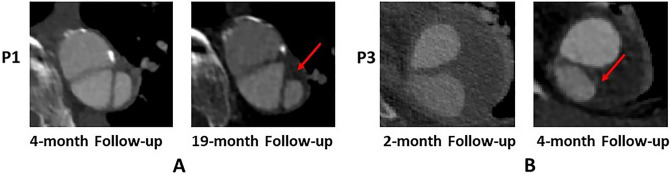
Partial thrombosis observed on CT scans in P1 (**A**) and P3 (**B**) at the subsequent follow-ups. Locations of thrombus formation are indicated by red arrows.

We performed CFD analysis on 4 cases of post-TEVAR dSINE in an attempt to examine hemodynamic differences between stable and unstable dSINEs. At first, low TAWSS and high RRT were observed on the surface of dSINE in both P1 and P3. High RRT indicates more flow stasis, which could enhance interactions between blood platelets and exposed tissue factors on the damaged wall, promoting thrombosis^22^. This is supported by the location of thrombus formation observed on post-SINE CT scans of P1 and P3 (Fig. 8). In P2 and P4, a large region of the SINE wall was observed with high TAWSS, and this region correlated with extremely low RRT (shown in Fig. 3c). Quantitative comparisons of RRT and TAWSS were made by averaging values over the entire SINE surface, and the results (Table 2) showed that P2 and P4 had an average TAWSS of 0.62 Pa and 0.42 Pa, respectively, which were much higher than P1 (0.17 Pa) and P3 (0.06 Pa). Additionally, P2 and P4 had a much lower average RRT of 3.5 and 6.7, respectively, compared to P1 (33.4) and P3 (87.5). In a similar study by Qiao et al.^18^, high RRT (> 5) was also identified as a strong predictor for non-deteriorating dSINEs, which is consistent with our finding. Our results provided further evidence that thrombosis occurred in areas of high RRT on the dSINE surface and high RRT is related to the shrinkage or stability of dSINEs by promoting thrombosis.

The association between TAWSS and dSINE progression was also examined. Based on a TAWSS threshold of 0.4 Pa—wall shear stress below this value could trigger atherosclerosis on the aortic wall^23^, Qiao et al. suggested that TAWSS alone was a weak predictor for the deterioration of dSINE^18^. Nevertheless, thrombosis instead of atherosclerosis is more likely to be a key factor influencing the stability of SINE. For thrombosis in the aorta, a lower TAWSS threshold (0.15 Pa) was needed to trigger coagulation and thrombosis^24,25^. This was further supported by the results presented in this study where P1 and P3 had an averaged TAWSS of 0.17 and 0.06 Pa, respectively, while P2 and P4 had a larger value of 0.62 and 0.42 Pa, respectively, in the dSINE. In addition, the location of SINE and its geometric shape may also affect TAWSS. Our study only included focal dSINEs confined to the distal end of the stent graft without distal connections with the FL. In future studies, a larger dataset will be involved, and the connection with the remaining FL will be further investigated.

The role of antegrade and retrograde flows in predicting FL expansion has been demonstrated in a recent retrospective study by Evangelista et al.^20^. They found high systolic antegrade flow volume in the FL and significant retrograde flow in diastole resulted in higher risk in FL expansion. In our study, similar findings were observed on dSINE expansion. The extent of retrograde flow was quantified by RFF at selected locations, and differences were observed between stable and unstable dSINEs. Plane 1 was placed around 10 mm above the dSINE and was far away from the aortic arch. At this location, there was very little retrograde flow in P1 and P3, but appreciable levels of retrograde flow were observed in P2 and P4, with RFF being 0.35 and 0.61%, respectively, in systole, and 0.26 and 0.45%, respectively, in diastole. On the SINE plane, considerable differences were observed with retrograde flow in P2 and P4 reaching 2.63 and 1.72%, respectively, in systole, and 4.37 and 3.49%, respectively, in diastole, compared to only 0.25 and 0.39% in P1, and 0.66 and 1.07% in P3 (Fig. 7 and Table 3). However, little difference was observed on plane 2, possibly due to the isolated nature of dSINE having limited influence on downstream flow. As dSINE expansion occurred in P2 and P4 at follow-up, our results indicate a potential role of RFF in predicting SINE expansion, and this parameter should be monitored in future studies.

In addition, SINE_*SA*_ and SINE_*DR*_ were calculated in a similar manner to Evangelista et al.’s study to quantify the effects of systolic SINE antegrade flow and diastolic SINE retrograde flow^20^. More systolic antegrade flows were observed in P2 (1.14%) and P4 (1.74%) compared with P1 (0.21%) and P3 (0.23%), but significant retrograde flows were observed in all patients during diastole (50.18, 50.64, 62.34 and 49.96% in P1, P2, P3 and P4, respectively), and these retrograde flows could be explained by the lack of additional connections between these isolated dSINEs and the FL. Our preliminary results suggest that high systolic antegrade flows in the SINE may be a potential predictor of dSINE expansion.

Finally, our current findings showed the potential predictive roles of hemodynamic metrics at an early stage of dSINE formation. As these parameters could also be measured or evaluated via 4D-flow magnetic resonance imaging (MRI)^20,26–32^, once fully validated, they could be used as biomarkers to assist in making accurate and timely decision about the need for a second intervention.

## Conclusion

This pilot study provided more insights into the hemodynamic details in dSINE and identified a potential role of hemodynamics in the stability of dSINEs. Our results showed that high RRT and low TAWSS may aid in dSINE shrinkage by promoting thrombosis, thus reducing the risk of dSINE expansion. Furthermore, we found that reverse flows quantified by RFF at the location of dSINE and high antegrade flows may indicate higher risks for dSINE expansion. These parameters should be monitored in future studies of a large patient cohort. Once validated, the identified parameters can be used to assist clinicians in planning treatment and determining the timing of additional intervention.

## Limitation

This study only included 4 patients who presented with an isolated dSINE in the descending aorta and an almost completely thrombosed FL after TEVAR. However, not all dSINEs are isolated and some may be connected to the remaining patent FL. Also, patient-specific boundary conditions were not available in this study, and the aortic wall was assumed to be rigid. The influences of patient-specific boundary conditions and wall compliance have been investigated previously^33–35^. Extending our methodology to a larger cohort will allow for investigation of a wider range of SINE morphologies and configurations. The assumption of rigid aortic wall is a key limitation. Previous research has shown that a rigid wall assumption could lead to overestimation of the size of low TAWSS region and less diverted flow^36,37^. However, since the region of interest in our study is the stented segment, wall motion will be significantly reduced due to the stiffness of the stent graft, which was observed in Qiao et al.’s fluid-structure interaction study^38^. Additionally, calcification and stiffening typically occur in chronic TBAD patients^39^, which further limits the movement of the stented region, therefore further reducing the impact of the rigid wall assumption in this study. As previous studies have demonstrated the importance of patient-specific inlet waveforms in simulations^33,40^, the lack of patient-specific waveform measurements affected flow measurements in this study. Specifically, stroke volume has been found to have a strong influence on wall shear stress and flow measurements^33^. The shape of the inlet waveform, such as the length of systole, would also affect the measurements of retrograde flows. Overall, as this study was designed to compare hemodynamic results between stable and unstable dSINEs, the consistence in boundary conditions across all patients allow for our comparative conclusions to be drawn. For clarity and further understanding, wall motion should be considered in the future to properly evaluate its impacts, and patient-specific boundary conditions should be applied to obtain more accurate results. Further validation of the hemodynamic results using patient-specific 4D-flow MRI data would be desirable to enhance the confidence of our findings.

## Methods

Four type B aortic dissection patients treated with TEVAR were included in this study, and a dSINE was detected in each patient at post-TEVAR follow-ups. Patient information and follow-up details are summarised in Table 4. The study complied with the Declaration of Helsinki and was approved by the ethics committee of Zhongshan Hospital, Fudan University. All patients provided written informed consent for participation. All experiments were performed in accordance with relevant guidelines and regulations. CT scans at the time when dSINE was first detected and at least 1 more post-SINE follow-ups were acquired. Patients 1 and 3 presented a stable dSINE with a reduction in volume, and patient 2 and 4 presented with recurrent back pain and a subsequent unstable dSINE with a rapid expansion in volume. All patients received medical treatments after the SINE was detected, and P4 received a second TEVAR after 97-month follow-up.

**Table 4:** Patient information on the first detection of SINE, the first post-SINE follow-up, the stability of SINE, and the size of the new tear.

	Gender	Age	First detection of SINE after TEVAR (month)	Post-SINE follow-up (month)	SINE stability	Tear size
P1	Male	66	4	19	Stable	0.95 cm^2^
P2	Male	72	72	75	Unstable	2.82 cm^2^
P3	Male	60	2	4	Stable	0.54 cm^2^
P4	Male	42	84	97	Unstable	3.07 cm^2^

Three-dimensional geometries were reconstructed from CT scans using Mimics (version 24.0; Materialise, Leuven, Belgium) based on a semi-automated, threshold-based algorithm. As the region of interest is the SINE, and all SINEs were observed in the thoracic region, each reconstructed geometry only involved the ascending aorta, aortic arch, and the thoracic descending aorta. The descending aorta was reconstructed up to 100 mm below the stent graft for patient 2, 3 and 4, and this distance was extended to 160 mm below the stent graft for patient 1 to include the remaining FL. Post-SINE geometries were also reconstructed. All geometries are shown in Fig. 1. The SINE volume was calculated as follows:

1 $$Vol_{SINE} = \mathop \sum \limits_{i = 1}^{N} \frac{{\left( {S_{i,SINE} + S_{i + 1,SINE} } \right)*h}}{2}$$

where S_*i*_, SINE is the cross-sectional area of the SINE for each axial slice i, N is the total number of axial slices, and h is the slice thickness. Percentage changes in SINE volume between the first SINE and post-SINE follow-up scans were calculated.

For each patient, the geometry extracted from the CT scan on which the SINE was first identified was used for CFD simulations. The four geometrical models were imported into ICEM CFD software (Ansys Inc, Canonsburg, PA, USA) and discretized into unstructured meshes consisting of a tetrahedral core and 10 prism wall layers. Mesh sensitivity tests were carried out on all geometries, and a maximum error of 5% in WSS was satisfied to ensure mesh-independent results. A final mesh consisting of approximately 3 million elements was used for each reconstructed geometry. As no patient-specific flow data was available, a pulsatile flow waveform from a type B aortic dissection patient presented in the literature was applied at the inlet for all simulations^33^. At all outlets, a 3-element Windkessel model was applied with parameters taken from the literature and tuned based on patient-specific pulse pressure measurements and geometrical characteristics^34^. Tuned parameters are shown in Table 5. The wall was assumed to be rigid, and the Bird-Carreau non-Newtonian model (Eq. 2) was used to account for shear-thinning properties of blood.

2 $$\mu = \mu_{\infty } + \left( {\mu 0 - \mu_{\infty } } \right)\left[ {{1} + (\lambda \dot{\gamma })^{{2}} } \right]^{{\left( {{\text{n}} - {1}} \right)/{2}}}$$

where the high-shear viscosity *µ*_∞_ = 0.0035 Pa s, the low-shear viscosity *µ*_0_ = 0.056 Pa s, the time constant *λ* = 3.313 s, and power law index n = 0.3568^41^. All simulations were performed using Ansys CFX (version 2020R2; Ansys Inc). A fixed timestep of 0.001 s was selected, and 5 cardiac cycles were simulated for each patient-specific model to ensure a periodic solution. Simulation results from the last cycle were used for analysis.

**Table 5:** Windkessel parameters for all model outlets.

	R1 (1e^8^ Pa s m^−3^)				C (1e^−9^ m^3^ Pa^−1^)				R2 (1e^8^ Pa s m^−3^)			
	P1	P2	P3	P4	P1	P2	P3	P4	P1	P2	P3	P4
IA	0.25	0.44	0.53	1.14	1.66	1.85	1.95	1.76	10.52	9.22	8.71	9.02
LCCA	0.86	2.44	2.5	–	0.57	0.42	0.5	–	30.63	40.59	33.34	–
LSA	–	0.96	1.53	–	–	0.93	0.77	–	–	18.22	21.86	–
Outlet	0.18	0.12	0.26	0.28	12.95	11.97	11.97	11.97	1.2	1.37	1.24	1.05

3-EWM parameters: R1: central resistance, C: compliance, R2: peripheral resistance; IA: Innominate artery; LCCA: Left common carotid artery; LSA: Left subclavian artery.

Time-averaged wall shear stress (TAWSS) and relative residence time (RRT) were calculated as defined in Eqs. (3) and (4) using post-processing software Ansys Ensight (version 2020R2; Ansys Inc), where oscillatory shear index (OSI) is related to the azimuthal variation of flow direction during the cardiac cycle and varies between 0 and 0.5. Antegrade and retrograde flows were measured at three different planes. Planes 1 and 2 were placed above and below the SINE, respectively, and a third plane (SINE plane) was placed at the transverse cross section of the SINE (Fig. 1). The locations of planes 1 and 2 were selected in a relative straight segment of the aorta to minimise the effects of curvature on flow distribution. Flow was quantified by calculating the retrograde flow fraction (RFF) during cardiac systole and diastole, respectively, systolic antegrade SINE flow fraction (SINE_*SA*_%) with respect to the total aortic (TL and SINE) systolic antegrade flow, and diastolic retrograde SINE flow fraction (SINE_*DR*_%) with respect to the total (retrograde and antegrade) diastolic SINE flow based on Eqs. (5, 6, 7). RFF, SINE_SA_ and SINE_DR_ are important parameters to evaluate systolic antegrade flows and diastolic retrograde flows which have been used to assess the risk of aorta and the FL enlargement in several studies^20,27,28^.

3 $$\begin{array}{*{20}c} {TAWSS = \frac{1}{T}\mathop \smallint \limits_{0}^{T} \left| {\tau \left( t \right)} \right|dt} \\ \end{array}$$

4 $$\begin{array}{*{20}c} {RRT = \frac{1}{{TAWSS\left( {1 - 2*OSI} \right)}}} \\ \end{array}$$

5 $$RFF = \frac{Retrograde\;Flow}{{Net\;Flow}}*100\%$$

6 $$SINE_{SA} = \frac{{{\text{Systolic}}\;{\text{SINE}}\;{\text{antegrade}}\;{\text{flow }}}}{{{\text{Systolic}}\;{\text{TL}}\;{\text{antegrade}}\;{\text{flow}} + {\text{Systolic}}\;{\text{SINE}}\,{\text{antegrade}}\;{\text{flow}}}}*100\%$$

7 $$SINE_{DR} = \frac{{{\text{Diastolic}}\;{\text{SINE}}\;{\text{retrograde}}\,{\text{flow}}}}{{{\text{Diastolic}}\;{\text{SINE}}\;{\text{antegrade}}\;{\text{flow }} + {\text{Diastolic}}\;{\text{SINE}}\;{\text{retrograde}}\,{\text{flow}}}}*100\%$$

### Informed consent statement

Informed consent was obtained from the subject involved in the study.

## Data Availability

The datasets generated and/or analysed during the current study are available from the corresponding authors on reasonable request.
